# CT Angiography or Cardiac MRI for Detection of Coronary Artery Aneurysms in Kawasaki Disease

**DOI:** 10.3389/fped.2021.630462

**Published:** 2021-02-04

**Authors:** Diana van Stijn, Nils Planken, Irene Kuipers, Taco Kuijpers

**Affiliations:** ^1^Department of Pediatric Immunology, Rheumatology and Infectious Diseases, Emma Children's Hospital, Amsterdam University Medical Center (UMC), University of Amsterdam, Amsterdam, Netherlands; ^2^Department of Radiology and Nuclear Medicine, Amsterdam University Medical Center (UMC), University of Amsterdam, Amsterdam, Netherlands; ^3^Department of Pediatric Cardiology, Emma Children's Hospital, Amsterdam University Medical Center (UMC), University of Amsterdam, Amsterdam, Netherlands

**Keywords:** Kawasaki disease, imaging, cardiac MRI, coronary computed tomographic angiography, coronary artery aneurysms, coronary artery assessment

## Abstract

**Background:** Kawasaki disease (KD) is an acute vasculitis that mainly affects the coronary arteries. This inflammation can cause coronary artery aneurysms (CAAs). Patients with KD need cardiac assessment for risk stratification for the development of myocardial ischemia, based on *Z-*score (luminal diameter of the coronary artery corrected for body surface area). Echocardiography is the primary imaging modality in KD but has several important limitations. Coronary computed tomographic angiography (cCTA) and Cardiac MRI (CMR) are non-invasive imaging modalities and of additional value for assessment of CAAs with a high diagnostic yield. The objective of this single center, retrospective study is to explore the diagnostic potential of coronary artery assessment of cCTA vs. CMR in children with KD.

**Methods and Results:** Out of 965 KD patients from our database, a total of 111 cCTAs (104 patients) and 311 CMR (225 patients) have been performed since 2010. For comparison, we identified 54 KD patients who had undergone both cCTA and CMR. CMR only identified eight patients with CAAs compared to 14 patients by cCTA. CMR missed 50% of the CAAs identified by cCTA.

**Conclusions:** Our single center study demonstrates that cCTA may be a more sensitive diagnostic tool to detect CAAs in KD patients, compared to CMR.

## Introduction

Kawasaki disease (KD) is an acute vasculitis of the medium-and-small-sized arteries of unknown etiology. To date, KD is the most common acquired pediatric heart disease in Western society (1). The vasculitis mainly affects the coronary arteries and the inflammation can cause coronary artery aneurysms (CAAs). Due to the formation of CAAs, CAA-related secondary complications can occur such as thrombosis, calcification, and stenosis/occlusion which can lead to myocardial ischemia. The occurrence of stenosis and thrombosis may well be inherent to the size of the CAA (2). Currently, echocardiography is used as the primary imaging modality in KD, and is a good first, rapid, and non-invasive screening tool in the acute phase. According to the American Heart Association (AHA) guidelines of 2017, additional imaging should be considered during follow-up after the patient has been categorized (by echocardiography) with a CAA (Z-score ≥ 2.5) (3), due to an associated increased risk for myocardial ischemia. This is where the Japanese Circulation Society (JCS) differs in their recommendations from the AHA. Low diagnostic accuracy of echocardiography due to limited visualization of distal coronary segments may result in underestimation of the CAA burden and may increase the risk for secondary complications (4). Therefore, the JCS suggests performing additional imaging in the convalescent phase (5) for a more accurate categorization of CAA severity.

Invasive Coronary Angiography (CAG), coronary computed tomographic angiography (cCTA) and Cardiac MRI (CMR) have been suggested as alternatives complementary to echocardiography by the AHA guidelines of 2017. Invasive CAG is not routinely used because of its invasive nature and risk of complications. In a recent overview, the need for guidance for the long-term management of KD patients was emphasized, suggesting non-invasive modalities such as echocardiography and CMR, and only when other modalities cannot be used, to consider low radiation dose computed tomography (CT) (6). As the limitations of echocardiography are known, we have been performing additional imaging such as cCTA and CMR, in selected patients in our national referral center (7). In our previous study we have demonstrated the relevance of routine additional imaging for coronary artery assessment by evaluating echocardiography and cCTA for the detection of CAAs, secondary coronary artery pathology, and radiation exposure (4). In the current study, we took the approach to investigate CAA detection with cCTA and CMR, if imaging results were both available in the same patient during follow-up. The aim of this retrospective single-center study is to compare the diagnostic yield of cCTA and CMR in clinical practice for the detection of CAAs in KD patients.

## Methods

### Study Population

Patients that met the AHA diagnostic criteria for KD, and presented to the follow-up of the national referral center for KD in the Netherlands and underwent CMR and cCTA between the year 2008 and 2020, were retrospectively included in this study. The AHA diagnostic criteria for KD are: persistent fever for ≥5 days and ≥4 of the five clinical features (rash, conjunctivitis, cervical lymphadenopathy, oral changes, and extremity changes) in the case of complete KD and, for incomplete KD, if fewer than 4 of the clinical features with prolonged unexplained fever and compatible echocardiography and/or laboratory findings are present. As cCTA and CMR are not part of the routine cardiac assessment in patients with KD, these patients are a selected subset of the KD population. The majority of these patients had been previously diagnosed with CAA upon echocardiography. It is exactly this group of patients (with proximal coronary artery pathology upon echocardiography) that has the risk for potentially missed distal coronary artery involvement upon echocardiography, since the patients with no proximal involvement have no reports on having distal involvement (4). The primary objectives for additional imaging in these patients were: i.e., to verify whether CAAs could be missed in the distal parts of the coronary arteries, beyond the window of inspection by echocardiography, and—at the same time—to look for secondary coronary artery pathology (such as stenosis, occlusion, calcification, or thrombus formation). Beta-blockers were used if patients were older than the age of 12 and had a heart rate above 70–75 beats per minute (BPM) prior to the imaging. Clinical information about the acute phase and of the follow-up was extracted from medical records. Institutional Review Board approval was obtained.

### CMR

Magnetic resonance imaging (MRI) images were acquired using a 1.5-T whole body MRI scanner with cardiac software (Siemens, Magnetom, Avanto; Siemens, Erlangen, and Germany). The imaging protocol included a navigator gated, ECG-triggered, non-contrast enhanced magnetic resonance coronary angiography (MRA) series, using a 3D echo time (TE)/repetition time (TR) optimized steady state free precession sequence with a fat saturated prepulse and T2 preparation (FOV 340–400 mm, base resolution 288 pixels. This resulted in a 3D image with a resolution of ~0.6 × 0.6 × 1.0 mm/pixel, encompassing the entire coronary tree. Acceptance window of the navigator was set to 2 mm. ECG triggering was set to the period of diastasis in the heart cycle. Imaging results were discussed in a multi-disciplinary team, consisting of a radiologist, cardiologist, pediatric cardiologist, and pediatric immunologist (all with expertise in KD).

### cCTA

For cCTA a dual-source 2 × 192-slice multidetector CT scanner (Siemens Somatom Force, Erlangen, Germany) was used from November 2015. Before 2015, the cCTA images were acquired using a 64-slice CT scanner (Philips, Brilliance64). For both scanners a prospective ECG-triggered step-and-shoot protocol was used and images were reconstructed with a slice-thickness of 0.6 and 0.9 mm, respectively. Contrast medium (Ultravist 300 mg/ml, Bayer Healthcare Pharmaceuticals) was administered intravenously. The total iodine dose and iodine delivery rate were adjusted for body weight. The scan delay was determined using a test bolus, after which 4 s were added for the scan delay of the main bolus. A multidisciplinary team including a radiologist, cardiologist, pediatric cardiologist, and pediatric immunologist (with expertise in KD) discussed the results, as reported previously (4).

### Measurements

Coronary artery diameters were measured and the CAAs *Z*-score was calculated according to the McCrindle/Boston model (8). There is no current alternative to the standardized measures of CAA obtained by echocardiography (9). Hence, we have used these values as the best available substrate, recognizing that they are obtained by measuring different components of the coronary arteries (internal appearance of wall to wall on echocardiography, rather than luminal diameter of contract on cCTA/CMR). A *Z-*score ≥ 3 was considered to be an aneurysm (as compared to a *Z-*score ≥ 2.5, which is normally used). By using a cut-off of a *Z*-score ≥ 3 instead of a *Z*-score ≥ 2.5 we aimed to increase specificity because a *Z*-score ≥ 2.5 in 1 coronary artery branch occurs in 0.6% of afebrile children and a Z-score ≥ 3.0 occurs in 0.1% (3). Also a study found that coronary artery dimensions in febrile children (non-KD) are larger than those in afebrile children, but smaller than in febrile KD patients (9). Even though not performed at the same age, majority of the imaging took place in the stable phase of the disease (i.e., more than 2 years after onset of disease) when remodeling is not expected anymore. Thereafter, discrepancies in outcome (number of detected CAAs) were considered the result of a lack of diagnostic accuracy. The left main coronary artery (LMCA), left anterior descending artery (LAD), right coronary artery (RCA), and circumflex (Cx) were evaluated. A CAA in the Cx was defined as luminal diameter ≥ 4.0 mm (10). Not only luminal dimensions were visualized, also myocardial ischemia, vascular stenosis, occlusion, vessel wall calcification and intravascular thrombosis were reported. Stenosis was defined as a narrowed lumen which influences the blood flow while an occlusion is a complete blockage of the lumen with no reserve flow. When on CMR, the coronary arteries were not visualized distinctly enough to make accurate and reliable measurements, coronary arteries were classified as NORMAL/ABNORMAL by two independent radiologists, blinded for the initial echocardiography and any additional imaging.

### Statistics

We generated demographic characteristics of KD patients who underwent both cCTA and CMR, presented as numbers with percentages and, where appropriate, with their mean or median and ranges (Table 1).

**Table 1:** Demographics and characteristics of 54 KD patients with imaging performed both by cCTA and CMR.

**Demographics**	***n* = 54**	**Remarks**
Male	*n* = 43	
Female	*n* = 11	
Age in years at onset KD (median, range)	3.1 (0.12–11.15)	Age-at-onset was unknown in two patients.
Missed diagnosis, no treatment	*n* = 7	No treatment (IVIG/prednisone) received in seven cases, of which two did receive ASA.
Day of treatment after onset of fever (median, range)	8 (4–26)	In 2 patients the day of treatment was unclear.
Treatment > 10 days after fever onset	*n* = 11	
Non-responder to 1st IVIG	*n* = 11	Persistent fever > 48 h after IVIG treatment.
ΔTime in years (time between CMR and cCTA) (median, range)	3 (0–7)	In 3 patients the cCTA was performed after the CMR. ΔTime in years (time between cCTA and CMR) for these patients was 1, 1, and 4 years.
**CAA** ***Z-*** **score acute stage**		
• *Z-*score > 10^*^ (giant)	*n* = 12	
• *Z*-score 3–10^*^ (small- to medium-sized aneurysms)	*n* = 13	
• *Z-*score <3^*^ (no aneurysm)	*n* = 23	
• Unknown	*n* = 6	

* *CAA status is based on prior echocardiography results in the acute phase of KD*.

## Results

### Study Population

We collected the cCTA and CMR results from 54 pediatric KD patients who had undergone both imaging techniques during the follow-up and we compared the imaging results retrospectively, of which nine were performed before 2015. All of the CMRs were executed prior to cCTA, except for three cases. The majority of the study population in which both cCTA and CMR scanning has been performed, was male (80%). The median age at onset of disease was 3.1 years (range 0.12–11.15). A total of 12 patients (22%) had giant aneurysms (*Z*-score ≥ 10) following the acute presentation with clinical KD (Table 1). Classic KD diagnosis presenting with ≥4 of the 5 principal clinical features was present in the majority of cases (74%), incomplete KD was present in a minority (20%), and in the remaining three patients (6%) the clinical features at the acute stage of the disease were unknown. Most patients had been treated adequately with oral acetylsalicylic acid (ASA) and high-dose intravenous immunoglobulin (IVIG) [once (67%), or twice (20%)], whereas a minority of cases (13%) was initially missed and did not receive any treatment. The difference in median age for cCTA when compared to CMR [16.5 years (1–59) vs. 12 years (0–57)], medians, and ranges), is in part explained by the earlier availability of the non-invasive CMR modality whereas the third generation dual-source cCTA only became available in 2015. Anesthesia was used in two patients for CMR as well as for cCTA because of their young age. The remaining patients were scanned while conscious and alert, medication to manage heart rhythm (i.e., beta-blockers) were not routinely used, only when the heart rate exceeded 70–75 beats per minute. The two patients with a history of coronary artery bypass grafting (CABG) were excluded from analysis.

### CAA Detection

With respect to the accuracy of coronary abnormalities we identified a total of 30 CAAs in 14 patients upon cCTA against 15 CAAs upon CMR in eight patients (Table 2). When the CAAs, visualized by cCTA were considered valid and true, the distribution of CAAs missed by CMR was as followed: four CAAs in the LMCA, three CAAs in the RCA, seven CAAs in the LAD, and one CAA in the Cx. There was no clear cut-off in diameter above which the CMR was able to detect the CAAs (Table 3), but predominantly determined by imaging quality overall instead.

**Table 2:** Total CAAs detected by cCTA vs CMR.

**Coronary artery**	**CAA on cCTA**	**CAA on CMR**
LMCA	7	3
RCA	12	7
LAD	10	3
Cx	1	0

**Table 3:** CAAs missed by CMR, with accompanying *Z-*scores calculated from the luminal diameters acquired by cCTA.

**LMCA in mm (*Z* score)**	**RCA in mm (*Z-*score)**	**LAD in mm (*Z-*score)**	**Cx in mm**
5 (1.25)^a^	4 (3.3)	4 (3.0)	7
6 (4.26)	7.4 (9.15)	4 (2.52)^b^	
	6 (3.88)	6.4 (7.69)	
		9 (29.92)	
		7 (6.52)	
		5 (3.07)	

a *Patient was overweight (BMI 35.1) which strongly affected the calculation of his Z-score*.

b *Borderline Z-score of 2.52, but with an irregular wall and an internal diameter of >1.5 times that of the adjacent segment, hence counted as CAA*.

In one patient, the CMR detected four CAAs in the RCA and one CAA in the Cx, while the cCTA detected only three CAAs in the RCA and a normal Cx. The delay between CMR and cCTA was 10 months. A month prior to the cCTA a CAG was performed, the results of the CAG were in concordance with the cCTA. Imaging in this patient was performed in the 1st year after onset of disease [also referred to as the dynamic phase (4, 11)] therefore, this discrepancy is most likely due to remodeling.

The CMR detected CAAs in three patients which actually had no CAAs in the coronary artery tree upon cCTA (neither in echocardiography) whereas the CMR detected a CAA in the LAD (1 patient) and in the Cx (2 patients). The delay between CMR and cCTA was 61, 67, and 59 months. However, imaging in these patients was performed long after onset of disease (>2 years), also referred to as the static phase (4), making the contribution of remodeling or normalization much less likely as the explanation for these discrepancies.

### cCTA and CMR; Logistics and Failure Rates

Diagnostic failure rates to accurately assess the presence of vascular lesions in these patients by either cCTA or CMR were 9.6% (a total of nine coronary arteries were failed to visualize in five patients) and 59.6% (a total of 108 coronary arteries were failed to visualize in 31 patients), respectively, when both modalities were compared with each other. The main reason that CMR data acquisition failed to accurately assess the presence of a vascular lesion and therefore 1 or more coronary arteries could not be interpreted, more often than in cCTA, was due to motion artifacts caused by (i.e.,) irregular breathing and insufficient image quality; all but two patients were subsequently assessed successfully by cCTA. The cCTA gave insufficient results in five cases (without anesthesia) because of motion artifact (4) and so-called “streaking” due to beam hardening and scatter (1). In three patients the coronary artery segments which were visualized insufficiently by cCTA could be assessed by CMR or echocardiography and were unaffected. In the other two patients the Cx was not visualized well-enough either by cCTA, CMR and echocardiography. These latter two patients had no history of CAAs in any of the other coronary branches though.

### CAA-Related Secondary Complications

Imaging by cCTA was able to detect additional vascular pathology in nine patients with coronary features such as calcification (*n* = 8), stenosis (*n* = 4), occlusion (*n* = 1) which were not observed by routine CMR. Coronary artery thrombosis (*n* = 3) was detected only once by CMR. In three patients CMR enabled us to identify myocardial infarction, cCTA revealed signs of myocardial infarction in two of these patients but with much less accuracy.

### Clinical Repercussions

**Figure 1:**
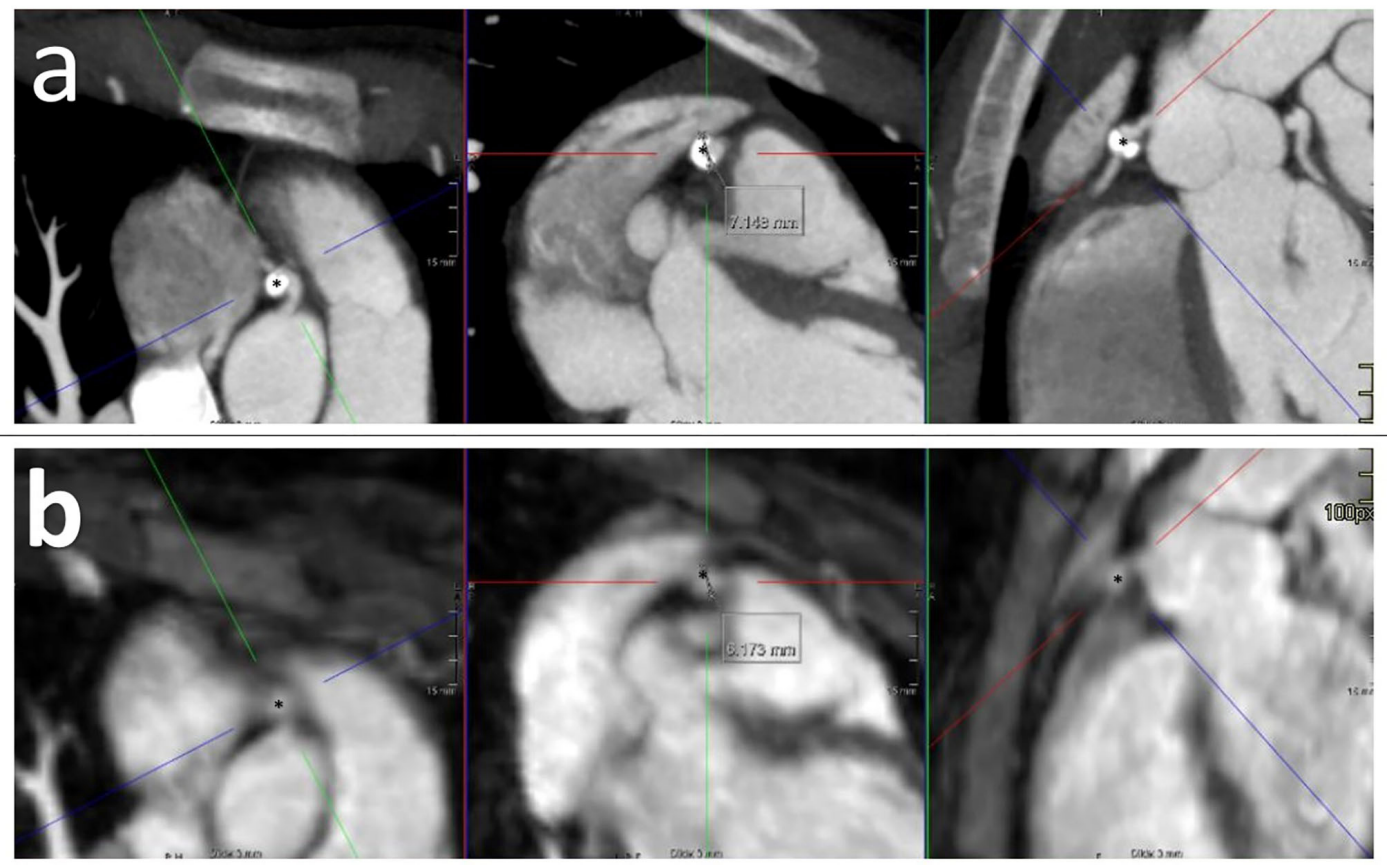
cCTA imaging **(a)** and CMR imaging **(b)** of KD patient with near giant CAA (*) in RCA. Calcification and partial coronary artery thrombus are only visible upon cCTA.

As a consequence of the insufficient performance of CMR, of the eight patients diagnosed with CAA upon CMR, four patients had a second or third CAA that were identified by cCTA and not by CMR. This did not lead to a change of CAA classification, in other words, the missed CAA did not exceed the other visible CAA in *Z*-score and therefore did not have clinical repercussions. CMR missed CAAs in three additional patients because of diagnostic failure (mainly due to motion artifact as mentioned before) which led to subsequent imaging by cCTA. Of these, one patient needed to start acetylsalicylic acid based on the new results of the cCTA. Finally, in the last three patients redefinition by cCTA of initially missed CAAs led to a different classification, i.e., from “no CAA” to “small CAA” in two patients and near giant CAA (*Z*-score 9.15) in one patient (Figure 1, Table 3). This last patient needed to start acetylsalicylic acid and also underwent subsequent CMR cardiac stress testing to detect possible myocardial damage, as she was pregnant at the moment of redefinition by cCTA, which showed no myocardial ischemia.

## Discussion

Our study in KD indicates that cCTA is the better modality to assess the coronary artery lesions in clinical practice when compared to CMR, and can be used for a more precise risk stratification and monitoring during follow-up of patients.

Third generation dual-source cCTA has proven to be of great value for the evaluation of the luminal diameter of the coronary artery compared to invasive coronary angiography in adults (12, 13) and in KD patients (14) as well as the detection of CAAs and CAA-related secondary complications in KD (4, 15–17). The strength of cCTA lies predominantly in visualizing the anatomy and therefore to detect aneurysms, stenosis, calcification, and thrombosis. Other benefits of the third dual-source cCTA are the low radiation exposure and fast acquisition time leading to a lower burden on the pediatric population. Therefore, third generation dual-source cCTA appears to be a good candidate for coronary artery assessment in KD, i.e., risk stratification for the development of myocardial ischemia. However, CMR does not expose patients to radiation, unlike CT imaging and CAG. There is consensus that radiation exposure (due to imaging), is associated with an increase in lifetime cancer risk, especially in children and should be kept to a minimum (18, 19). Recent guidelines consider this radiation exposure and therefore mention CMR as a good imaging technique in KD (6), however, our data suggest cCTA is the more preferred modality for the visualization vascular morphology, thrombus formation and calcified lesions. CMR is valuable to visualize tissue characteristics and physiology, and has been used to demonstrate and in particular ischemia and tissue damage following myocardial infarction in KD (20, 21). Other imaging methods to evaluate cardiac function are being investigated as well (22). Current development in CT scanning techniques may enable cardiac function assessment in the future at low-dose radiation exposure (23, 24), but to date however, CMR remains the most suitable and best imaging modality for the evaluation of cardiac function and for detection of ischemia and infarction.

Differences in applicability and accuracy between CMR and cCTA have been described in the past in adults with coronary artery disease (CAD) (25). These patients mainly presented with obstructive CAD due to plaque formation and may not be comparable with our pediatric population with KD. Previous studies have attempted to determine the clinical applicability of either CT or CMR for the risk stratification (4, 12–17, 21, 26–29), but there are no comparative imaging studies available for KD to date.

Upon comparison of both modalities in daily practice, our data shows that cCTA outperforms CMR in the detection of CAAs. CMR showed a higher diagnostic failure rate for coronary artery assessment mainly due to motion artifacts caused by protracted acquisition time, compared to cCTA (respectively, 59.6 vs. 9.6%). To note, suboptimal images were rated as “diagnostic failure,” as these suboptimal images led to the under-reporting of CAAs. The CAAs missed by CMR were more frequently localized in the LAD, followed by the LCA and RCA; of which three were missed because of inadequate performance.

Because echocardiography also had not been able to detect these CAAs, two patients went undertreated for 5 and 2 years, until their medium-sized CAAs were detected and oral medication was (re)started. In four patients, the CAA classification changed from normal to “small CAA,” which needed no further medication. The four patients in whom additional CAAs were detected that had been missed upon CMR, were already taking oral aspirin, but will be monitored by repeated cCTA during follow-up as a consequence. Important factors that contribute in the higher CAA detection rate by cCTA are high spatial resolution of the cCTA (0.6 mm/pixel vs. 0.6 × 0.6 × 1 mm/pixel in MRI), and high temporal resolution. This higher spatial resolution also contributes to the detection of additional coronary artery pathology (i.e., calcification, stenosis, and thrombosis), while CMR was inaccurate or unable to do so. These CAA-related secondary complications are relevant to be properly diagnosed for treatment considerations during follow-up. Another important factor for accurate coronary artery assessment is heart rate. A higher heart rate is a disadvantage for accurate coronary artery assessment. In our study, the average age of the children undergoing cCTA, and as a consequence their heart rate (since younger children on average have a higher heart rate than older children), was higher than in those that were imaged by CMR. Instead of the heart rate at start, longer acquisition time, and variability in heart rate upon CMR is probably the reason for a higher failure rate and lack of sufficient accuracy in CAA detection in KD. Stricter regulation of heart rate, managed by beta-blockers, could improve the diagnostic accuracy of CMR.

### Limitations

Retrospective analysis introduces variation of the data, having not been systematically collected in a predefined prospective manner. In our case, patients have been selected based on the prerequisite of having complementary imaging with both CMR and cCTA data available in the same patients. Most often these patients were known to have CAAs as previously visualized in the (sub)acute stage upon echocardiography. Thus, our patient cohort does not represent a normal unselected KD population. This, however, may not be a disadvantage because it is exactly this subgroup of patients that should be routinely monitored more closely. Despite the fact that most imaging was performed in the stable phase in which remodeling of coronary artery lesions is not expected anymore, the delay between both imaging techniques may have been of influence on our results. Since there were no luminal diameters available for some of the coronary arteries on CMR, the arteries were scored as “normal” or “abnormal” based on the experienced eye. This approach was considered the most realistic for subsequent comparisons between the two imaging groups (i.e., cCTA and CMR). The inability to measure the exact coronary artery diameter on CMR supports our first suspicion that CMR could have a lower diagnostic accuracy compared to cCTA.

Finally, we calculated the *Z*-scores using the McCrindle/Boston model, although not formally validated for being used for imaging by cCTA and CMR.

## Conclusion

Our study in KD shows that cCTA is an excellent imaging modality to assess the coronary artery tree at great resolution. cCTA detects CAAs more frequent and with greater detail when compared to CMR. Therefore, we recommend to perform cCTA in addition to echocardiography in CAA positive KD patients to detect and classify CAAs.

## Data Availability Statement

The original contributions presented in the study are included in the article/supplementary material, further inquiries can be directed to the corresponding author/s.

## Ethics Statement

Ethical review and approval was not required for the study on human participants in accordance with the local legislation and institutional requirements. Written informed consent to participate in this study was provided by the participants' legal guardian/next of kin.

## Author Contributions

DS conceptualized the study, collected data, and drafted the initial manuscript. IK and TK contributed equally as co-senior authors and conceptualized the study, coordinated, and supervised data collection and reviewed the manuscript for important intellectual content and revised the manuscript. NP conceptualized the study and reviewed for important intellectual content and revised the manuscript. All authors approved the final manuscript as submitted and agree to be accountable for all aspects of the work.

## Conflict of Interest

The authors declare that the research was conducted in the absence of any commercial or financial relationships that could be construed as a potential conflict of interest.

## Abbreviations

AHA: American Heart Association
ASA: acetylsalicylic acid
BPM: beats per minute
CAAs: Coronary artery aneurysms
CABG: coronary artery bypass grafting
CAD: coronary artery disease
CAG: coronary angiography
cCTA: coronary computed tomographic angiography
CMR: cardiac magnetic resonance imaging
CT: computed tomography
Cx: circumflex
IVIG: intravenous immunoglobulin
JCS: Japanese Circulation Society
KD: Kawasaki disease
LAD: left anterior descending artery
LMCA: left main coronary artery
MRA: magnetic resonance coronary angiography
MRI: magnetic resonance imaging
RCA: right coronary artery
TE: echo time
TR: repetition time.

## References

[1] Kato H Koike S Yamamoto M Ito Y Yano E. Coronary aneurysms in infants and young children with acute febrile mucocutaneous lymph node syndrome. J Pediatr. (1975) 86:892–8. 10.1016/S0022-3476(75)80220-4 236368

[2] McCrindle BW Manlhiot C Newburger JW Harahsheh AS Giglia TM Dallaire F. Medium-term complications associated with coronary artery aneurysms after kawasaki disease: a study from the International Kawasaki Disease Registry. J Am Heart Assoc. (2020) 9:e016440. 10.1161/JAHA.119.016440 32750313 PMC7792232

[3] McCrindle BW Rowley AH Newburger JW Burns JC Bolger AF Gewitz M. Diagnosis, treatment, and long-term management of Kawasaki disease: a scientific statement for health professionals from the American Heart Association. Circulation. (2017) 135:e927–e99. 10.1161/CIR.0000000000000484 28356445

[4] van Stijn-Bringas Dimitriades D Planken RN Groenink M Streekstra GJ Kuijpers TW Kuipers IM. Coronary artery assessment in Kawasaki disease with dual-source CT angiography to uncover vascular pathology. Eur Radiol. (2019) 30:432–41. 10.1007/s00330-019-06367-6 31428828 PMC6890577

[5] Fukazawa R Kobayashi J Ayusawa M Hamada H Miura M Mitani Y. JCS/JSCS 2020 guideline on diagnosis and management of cardiovascular sequelae in Kawasaki disease. Circ J. (2020) 84:1348–1407. 10.1253/circj.CJ-19-1094 32641591

[6] Brogan P Burns JC Cornish J Diwakar V Eleftheriou D Gordon JB. Lifetime cardiovascular management of patients with previous Kawasaki disease. Heart. (2020) 106:411–20. 10.1136/heartjnl-2019-315925 31843876 PMC7057818

[7] Tacke CE Kuipers IM Biezeveld MH Groenink M Breunis WB Kuijpers TW. [Kawasaki disease: description of a Dutch cohort of 392 patients]. Ned Tijdschr Geneeskd. (2011) 155:A2698. 21418701

[8] McCrindle BW Li JS Minich LL Colan SD Atz AM Takahashi M. Coronary artery involvement in children with Kawasaki disease: risk factors from analysis of serial normalized measurements. Circulation. (2007) 116:174–9. 10.1161/CIRCULATIONAHA.107.690875 17576863

[9] Muniz JC Dummer K Gauvreau K Colan SD Fulton DR Newburger JW. Coronary artery dimensions in febrile children without Kawasaki disease. Circ Cardiovasc Imaging. (2013) 6:239–44. 10.1161/CIRCIMAGING.112.000159 23357243

[10] Newburger JW Takahashi M Gerber MA Gewitz MH Tani LY Burns JC. Diagnosis, treatment, and long-term management of Kawasaki disease: a statement for health professionals from the Committee on Rheumatic Fever, Endocarditis and Kawasaki Disease, Council on Cardiovascular Disease in the Young, American Heart Association. Circulation. (2004) 110:2747–71. 10.1161/01.CIR.0000145143.19711.78 15505111

[11] Dietz SM Kuipers IM Koole JCD Breur J Fejzic Z Frerich S. Regression and complications of z-score-based giant aneurysms in a Dutch cohort of Kawasaki disease patients. Pediatr Cardiol. (2017) 38:833–9. 10.1007/s00246-017-1590-0 28236162 PMC5388726

[12] Nieman K Cademartiri F Lemos PA Raaijmakers R Pattynama PM de Feyter PJ. Reliable noninvasive coronary angiography with fast submillimeter multislice spiral computed tomography. Circulation. (2002) 106:2051–4. 10.1161/01.CIR.0000037222.58317.3D 12379572

[13] Ropers D Baum U Pohle K Anders K Ulzheimer S Ohnesorge B. Detection of coronary artery stenoses with thin-slice multi-detector row spiral computed tomography and multiplanar reconstruction. Circulation. (2003) 107:664–6. 10.1161/01.CIR.0000055738.31551.A9 12578863

[14] Tsujii N Tsuda E Kanzaki S Kurosaki K. Measurements of coronary artery aneurysms due to Kawasaki disease by dual-source computed tomography (DSCT). Pediatr Cardiol. (2016) 37:442–7. 10.1007/s00246-015-1297-z 26515298

[15] Duan Y Wang X Cheng Z Wu D Wu L. Application of prospective ECG-triggered dual-source CT coronary angiography for infants and children with coronary artery aneurysms due to Kawasaki disease. Br J Radiol. (2012) 85:e1190–7. 10.1259/bjr/18174517 22932064 PMC3611723

[16] Yu Y Sun K Wang R Li Y Xue H Yu L. Comparison study of echocardiography and dual-source CT in diagnosis of coronary artery aneurysm due to Kawasaki disease: coronary artery disease. Echocardiography. (2011) 28:1025–34. 10.1111/j.1540-8175.2011.01486.x 21854436

[17] Singhal M Singh S Gupta P Sharma A Khandelwal N Burns JC. Computed tomography coronary angiography for evaluation of children with Kawasaki disease. Curr Probl Diagn Radiol. (2018) 47:238–44. 10.1067/j.cpradiol.2017.09.013 29203262

[18] Preston DL Ron E Tokuoka S Funamoto S Nishi N Soda M. Solid cancer incidence in atomic bomb survivors: 1958-1998. Radiat Res. (2007) 168:1–64. 10.1667/RR0763.1 17722996

[19] Task Group on Control of Radiation Dose in Computed T. Managing patient dose in computed tomography. A report of the International Commission on Radiological Protection. Ann ICRP. (2000) 30:7–45. 10.1016/S0146-6453(01)00049-5 11711158

[20] Tsuda E Singhal M. Role of imaging studies in Kawasaki disease. Int J Rheum Dis. (2017) 22:7–45. 10.1111/1756-185X.13210 29115035

[21] Tacke CE Kuipers IM Groenink M Spijkerboer AM Kuijpers TW. Cardiac magnetic resonance imaging for noninvasive assessment of cardiovascular disease during the follow-up of patients with Kawasaki disease. Circ Cardiovasc Imaging. (2011) 4:712–20. 10.1161/CIRCIMAGING.111.965996 21921132

[22] Dedeoglu R Barut K Oztunc F Atik S Adrovic A Sahin S. Evaluation of myocardial deformation in patients with Kawasaki disease using speckle-tracking echocardiography during mid-term follow-up. Cardiol Young. (2017) 27:1377–85. 10.1017/S1047951117000580 28376935

[23] Savino G Zwerner P Herzog C Politi M Bonomo L Costello P. CT of cardiac function. J Thorac Imaging. (2007) 22:86–100. 10.1097/RTI.0b013e3180317433 17325580

[24] Groves DW Olivieri LJ Shanbhag SM Bronson KC Yu JH Nelson EA. Feasibility of low radiation dose retrospectively-gated cardiac CT for functional analysis in adult congenital heart disease. Int J Cardiol. (2017) 228:180–3. 10.1016/j.ijcard.2016.11.108 27865183 PMC6323633

[25] Nikolaou K Alkadhi H Bamberg F Leschka S Wintersperger BJ. MRI and CT in the diagnosis of coronary artery disease: indications and applications. Insights Imaging. (2011) 2:9–24. 10.1007/s13244-010-0049-0 22347932 PMC3259311

[26] Schuijf JD Bax JJ Shaw LJ de Roos A Lamb HJ van der Wall EE. Meta-analysis of comparative diagnostic performance of magnetic resonance imaging and multislice computed tomography for noninvasive coronary angiography. Am Heart J. (2006) 151:404–11. 10.1016/j.ahj.2005.03.022 16442907

[27] Takemura A Suzuki A Inaba R Sonobe T Tsuchiya K Omuro M. Utility of coronary MR angiography in children with Kawasaki disease. AJR Am J Roentgenol. (2007) 188:W534–9. 10.2214/AJR.05.1414 17515343

[28] Greil GF Seeger A Miller S Claussen CD Hofbeck M Botnar RM. Coronary magnetic resonance angiography and vessel wall imaging in children with Kawasaki disease. Pediatr Radiol. (2007) 37:666–73. 10.1007/s00247-007-0498-x 17541574

[29] Bluemke DA Achenbach S Budoff M Gerber TC Gersh B Hillis LD. Noninvasive coronary artery imaging: magnetic resonance angiography and multidetector computed tomography angiography: a scientific statement from the american heart association committee on cardiovascular imaging and intervention of the council on cardiovascular radiology and intervention, and the councils on clinical cardiology and cardiovascular disease in the young. Circulation. (2008) 118:586–606. 10.1161/CIRCULATIONAHA.108.189695 18586979

